# Study on the Charge Characteristics and Migration Characteristics of Amorphous Alloy Core Debris

**DOI:** 10.3390/ma18184415

**Published:** 2025-09-22

**Authors:** Wenxu Yu, Xiangyu Guan

**Affiliations:** College of Electrical Engineering and Automation, Fuzhou University, Fuzhou 350108, China; xiangyuguan1986@163.com

**Keywords:** amorphous alloy core debris, migration characteristic, charge characteristics, lattice Boltzmann method, discrete element method

## Abstract

Compared with a traditional distribution transformer with silicon steel sheet as the core material, the no-load loss of an amorphous alloy transformer is greatly reduced due to its core using iron-based amorphous metal material, which has been applied in many countries. However, due to the brittleness of its amorphous strip, an amorphous alloy transformer is prone to debris in the process of production, transportation and work. The charge and migration characteristics of these debris will reduce the insulation strength of the transformer oil and endanger the safe operation of the transformer. In this paper, a charge measurement platform of amorphous alloy debris is set up, and the charging characteristics of amorphous alloy core debris under different flow velocities, particle radius and plate electric field strength are obtained. The results show that with an increase in pipeline flow velocity, the charge-to-mass ratio of the debris increases first and then decreases. With an increase in electric field strength, the charge-to-mass ratio of the debris increases; with an increase in the number of debris, the charge-to-mass ratio of the debris decreases; with an increase in debris size, the charge-to-mass ratio of the debris increases. The debris with different charge-to-mass ratios and types obtained from the above experiments are added to the simulation model of an amorphous alloy transformer. The lattice Boltzmann method (LBM) coupled with the discrete element method (DEM) is used to simulate the migration process of metal particles in an amorphous alloy transformer under the combined action of gravity, buoyancy, electric field force and oil flow resistance under electrothermal excitation boundary. The results show that the trajectory of the debris is related to the initial position, electric field strength and oil flow velocity. The LBM–DEM calculation model and charge measurement platform proposed in this paper can provide a reference for studying the charge mechanism and migration characteristics of amorphous alloy core debris in insulating oil.

## 1. Introduction

The amorphous alloy transformer is widely used in power systems because its core is made of amorphous alloy strip. Compared with a core transformer made of traditional silicon steel material, it has the advantage of greatly reducing no-load loss [1,2,3,4]. Despite the considerable economic and environmental advantages offered by amorphous alloy transformers, the inherent limitations of amorphous materials pose significant operational challenges. First, compared to silicon steel, amorphous alloys exhibit a stronger magnetostriction coefficient, leading to more pronounced core vibrations under alternating magnetic flux. Since an amorphous core cannot be excessively compressed, its weakened vibration restraint further exacerbates transformer noise. Second, amorphous alloys are highly sensitive to mechanical stress; excessive external force on the core severely degrades its magnetic permeability and results in a sharp increase in core loss. Finally, annealing is essential for amorphous alloy ribbons to achieve optimal low-loss characteristics, but this treatment also increases their brittleness [5]. Consequently, during transformer production, transportation, assembly and operation, abnormal vibrations may easily generate amorphous alloy debris from the core [6]. The debris will migrate under the action of the electric field and the flow field, and the charge will also be transferred, which will distort the electric field and reduce the insulation strength of the oil gap, thus seriously affecting the service life and operation status of the power equipment [7]. Therefore, it is necessary to study the charge characteristics and migration characteristics of amorphous alloy core debris in insulating oil.

At present, the research on amorphous alloy transformers mostly focuses on its core materials, such as magnetic properties and vibrations. For example, W. Zhang et al. studied the magnetic properties of different materials such as nanocrystals and amorphous alloys at different frequencies and temperatures [8]. Y. Li et al. developed a new type of iron-based nanocrystals, which can be directly fabricated by atomization and have low hysteresis loss [9]. R. K. Noubissi et al. established a three-dimensional model to study the vibration of an amorphous alloy transformer core [10]. Although improving the magnetic properties of the core material and reducing the vibration can reduce the generation of amorphous alloy core debris, it cannot be avoided. At the same time, in the process of transportation and production, the insulating oil may also be contaminated by debris. In order to explore the tolerance of insulating oil, a large number of scholars have carried out related research, but most of them focus on the study of ions, bubbles, fibers and metal particle impurities. For example, K Liang et al. studied the dielectric properties, breakdown properties and partial discharge characteristics of copper ions in transformer insulating oil and obtained the dielectric loss at different frequencies and the influence of different copper ion concentrations on the performance of insulating oil [11]. P Wang et al. studied the bridging characteristics of fiber particles in insulating oil under DC electric field. Under different electric fields and different mass fractions, the difficulty of bridging between electrodes is different, and it is analyzed from the perspective of the force of fiber particles [12]. However, there are few studies on the charge characteristics of amorphous alloy core debris in insulating oil and their migration characteristics under the action of electro–thermal–fluid coupling field.

The evolution of the lattice Boltzmann method has been instrumental in advancing heat and mass transfer and multiphase flow studies [13,14,15,16]. As an explicit numerical solution algorithm, the LBM has a natural advantage in solving the internal flow field of insulating oil [17]. Some scholars combined the LBM with the DEM to study the particle motion in multi-physical fields and simulated the motion law of different particles [18,19,20]. However, the LBM–DEM does not consider the charging mechanism of the particles themselves in the study of the migration characteristics of the particles, and most scholars calculate the flow field solved by the LBM and the particle motion solved by the DEM separately, without considering the influence of the reaction force of the drag force of the particle fluid on the fluid flow. Therefore, it is difficult to explore the charging characteristics and migration characteristics of particles of different sizes under complex conditions.

In this paper, an amorphous alloy transformer and its internal amorphous alloy core debris are taken as the research object, and a debris charging platform is built to explore the charging characteristics of debris under different flow rates, debris sizes and plate electric field strengths. At the same time, a numerical simulation model of an amorphous alloy transformer based on the LBM–DEM is constructed. Based on this model, the migration characteristics of amorphous alloy core debris in insulating oil under the action of electro-thermal-fluid coupling field are studied, which provides a method and reference for further exploring the influence of debris on insulating oil.

## 2. Charge Characteristic Measurement Experiment

### 2.1. The Construction of Physical Experiment Platform

**Figure 1:**
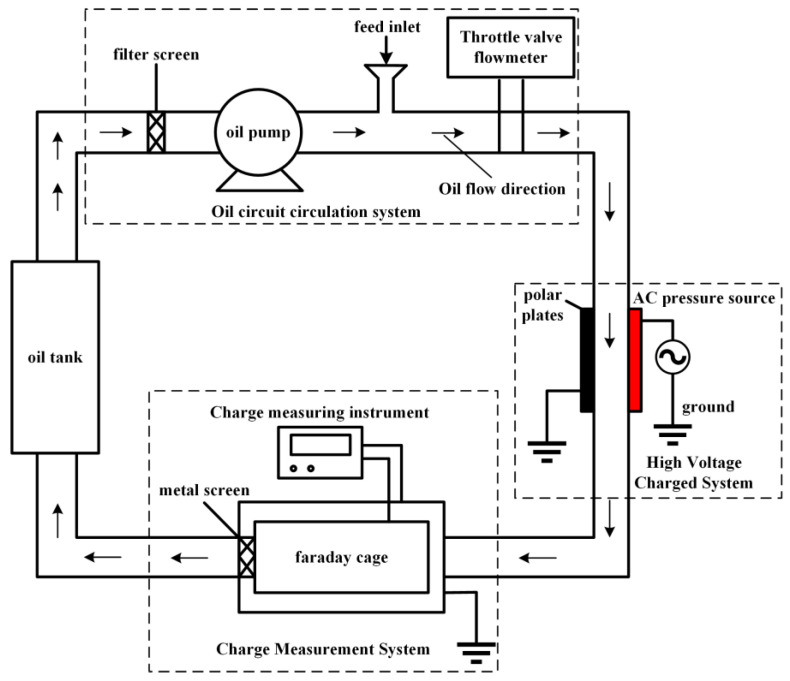
Metal particle charge measurement platform.

Since the amorphous alloy core debris in insulating oil will be affected by the operation of power equipment, that is, the amount of charge carried by the oil flow and the induction or contact of the charged body in the electric field due to the difference in oil temperature, the relative motion friction between the insulating oil and the debris will also dissociate the oil molecules of the debris and make the debris charged. In order to explore the charge characteristics of amorphous alloy core debris in insulating oil under different oil flow velocities, size and plate electric field strengths, a charge measurement platform is designed and built in this paper, as shown in Figure 1.

The test platform mainly includes the oil tank, the oil circulation system, the high-voltage charging system and the charge measurement system. Among them, the oil circulation system includes the oil pump, an insulated pipeline, a throttle valve and a flowmeter. The round gear flowmeter is used here. This flowmeter can measure the liquid flow with high viscosity, and then calculate the liquid flow rate. The high voltage charging system is mainly composed of two rectangular copper plate electrodes. The black plate is the grounding electrode, and the red plate is connected to the AC high voltage. By applying the AC electric field, the electric field environment inside the transformer oil channel is simulated. The charge measurement system is mainly composed of a Faraday cup and a charge-measuring instrument. The Faraday cup is composed of two coaxially connected metal cylinders. The inner cylinder is used to sense the charge of the debris, and the outer cylinder is used to ground and shield the external electromagnetic interference and filter out the charge in the air. Through this platform, the charging characteristics of amorphous alloy core debris were measured, and the charging mechanism of debris was studied, which provided a basis for studying the migration characteristics of amorphous alloy core debris in insulating oil.

### 2.2. Experimental Conditions and Measurement Process

**Figure 2:**
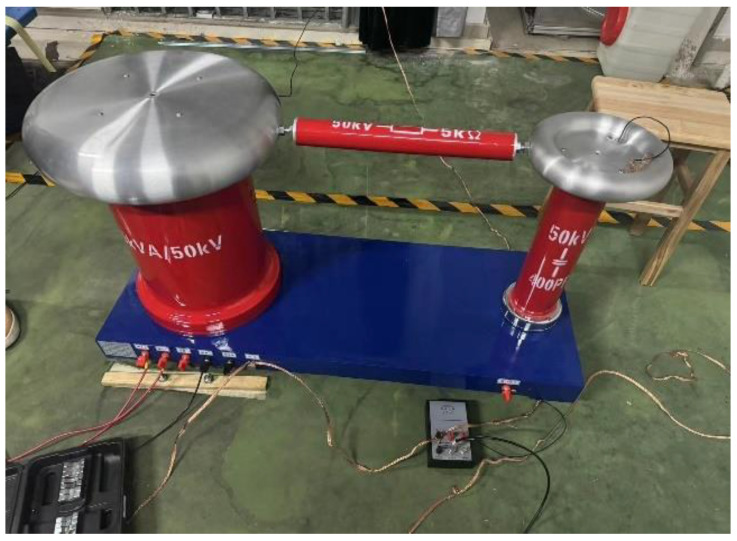
Non-partial discharge transformer.

In the experiment, the oil tank is first filled with filtered insulating oil (using No.25 transformer oil commonly used in power equipment), then the oil pump is opened, and the flow rate is adjusted. After reaching the set flow rate, the oil pump is closed, and the insulating oil is kept stationary. Secondly, amorphous alloy core debris (made of 1k101 iron-based amorphous alloy strip) of different radii are added to the funnel, and AC voltage is applied to the parallel plates of the high-voltage charging system by using the power frequency 50 kV non-partial discharge transformer (Figure 2). Then open the oil pump, and start the oil circulation system so that the insulating oil flows at a preset speed and the amorphous alloy core debris then flow to the plate gap and obtain a certain charge and enter the Faraday cup. The metal filter can ensure that all debris are collected in the Faraday cup. Finally, when the debris are collected, the power supply is disconnected, and the amount of charge carried by the debris can be measured by the charge tester (The Keithley 6517B electrometer is manufactured by Keithley Instruments, Solon, OH, USA). The physical parameters of the insulating oil and debris are shown in Table 1.

### 2.3. Experimental Result

The basic conditions of the experiment are as follows: the number of debris is 4, the size is 0.6 mm × 0.6 mm, the plate voltage is 6 kV, and the oil flow velocity in the pipeline is 0.16 m/s. The following experimental results are the results of debris charge and charge–mass ratio after multiple measurements when other conditions remain unchanged and only the corresponding variables are changed.

Firstly, the pipeline oil flow velocities are changed to 0.07 m/s, 0.12 m/s, 0.16 m/s, 0.19 m/s and 0.21 m/s, respectively (based on the simulation results of flow velocity field). The measurement results are shown in Table 2. It can be seen that when only the flow rate is changed, the charge amount *q* and charge-to-mass ratio *Φ* will increase first and then decrease. This phenomenon occurs because, as the flow rate increases, the relative motion between particles and the insulating oil intensifies, leading to enhanced frictional electrification and a corresponding rise in charge generation. Concurrently, an increased number of free oil molecules adhere to the amorphous alloy debris, further elevating their net charge. However, when the flow rate exceeds a certain threshold, the residence time of the debris within the plate decreases significantly. Additionally, the higher flow velocity reduces the likelihood of lateral contact or collision between the debris and the metal plate under the influence of the electric field. Consequently, the charge acquisition by the debris diminishes.

Secondly, the AC voltage levels are changed to 2 kV, 4 kV, 6 kV, 8 kV and 10 kV, respectively. The measurement results are shown in Table 3. It can be seen that with an increase in voltage, the charge and charge–mass ratio of particles will gradually increase. This is because an increase in voltage will increase the electric field strength, and the amount of charge induced by particles between the plates will increase. At the same time, due to the increase in electric field force, the collision probability between particles and plates will increase, and the amount of charge obtained will increase.

The number of debris was changed again to 4, 8 and 12, respectively. The measurement results are shown in Table 4. It can be seen that with an increase in the number of debris, the charge of the debris will gradually increase, but the charge-to-mass ratio will decrease. This is because the number of debris increases. Although the possibility of obtaining a charge for each debris is not much different, due to the collision between the debris or the collision with the plate, it is possible to make the polarity of the charge carried by the debris different so that part of the charge is neutralized. The Faraday cup measures the net charge. Therefore, as the debris increase, the increase in the total charge will slow down, and the charge-to-mass ratio will decrease.

The radii of the metal particles were changed to 0.2 mm, 0.4 mm, 0.6 mm, 0.8 mm and 1.0 mm, respectively. The measurement results are shown in Table 5. It can be seen that as the particle radius increases, the charge amount and charge-to-mass ratio of the particles will gradually increase. This is because the particle radius increases, making its surface area increase, and it is more prone to collision and friction to generate more charges.

## 3. LBM–DEM Coupling Modeling Analysis

**Figure 3:**
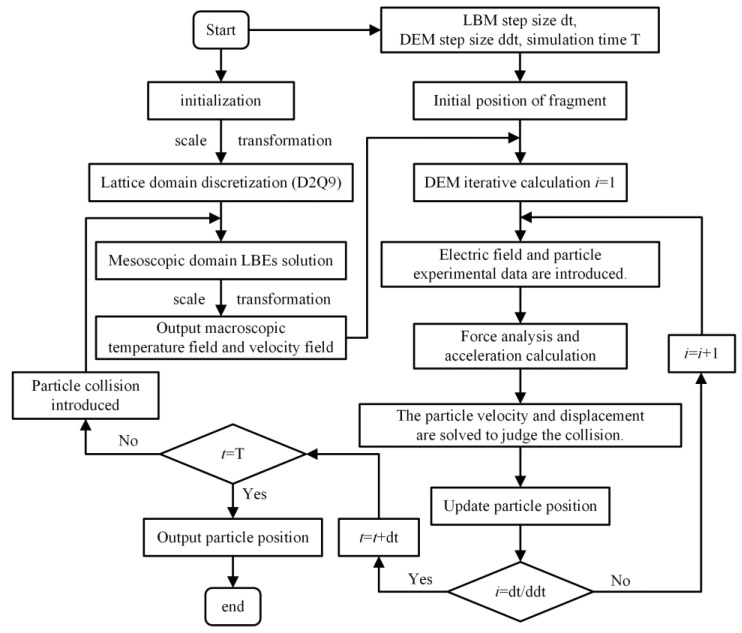
LBM–DEM calculation process.

The LBM is a method based on the molecular kinetic theory at the mesoscopic scale, which uses the distribution function to describe macroscopic physical quantities [21]. The main steps for solving the thermal-flow field include the discretization of the lattice domain, the solution of the lattice Boltzmann equations (LBEs) in the mesoscopic domain and the conversion of the macroscopic physical field. The DEM takes particles as discrete elements in the Lagrange coordinate system, discretizes time into a fixed micro time domain, divides space into micro space domains and combines Newton’s second law to establish equations to calculate the velocity and displacement of particles. In this paper, the LBM and DEM are coupled by an autonomous programming code in MATLAB R2023a, and the solution model of the LBM–DEM is established. Considering the force of particles on insulating oil, the migration characteristics of particles and the temperature field and velocity field inside the fluid are calculated. The solution process is shown in Figure 3.

### 3.1. Solution of Thermal-Flow Field Based on LBM

**Figure 4:**
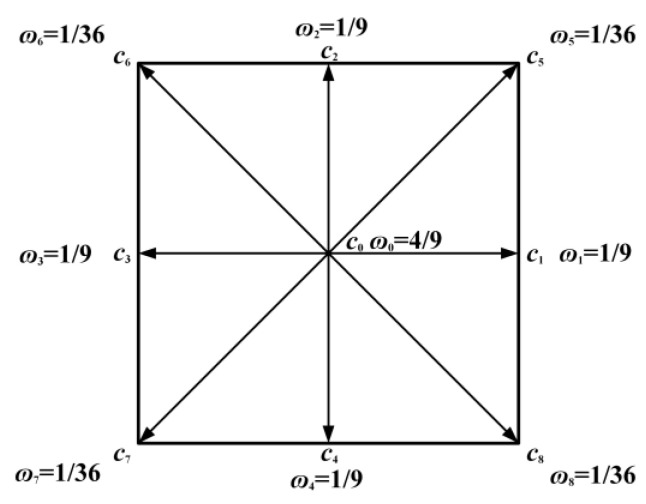
D2Q9 lattice model.

For the LBM solution, it is necessary to discretize the lattice domain first. In the lattice domain, D*x*Q*y* is generally used to represent the solution unit, where *x* represents the dimension and *y* represents the migration direction of the lattice. Therefore, based on the simplified double-distribution function (DDF) in Reference [22], the D2Q9 lattice model is used to solve the LBEs in the mesoscopic domain to describe the flow and heat transfer process. The specific migration direction and weight are shown in Figure 4.

The concrete expression of discrete velocities ***c_i_*** is defined as follows:

(1) $$c_{i}=\left\{\begin{array}{ll} \left(0,0\right) & i=0\\ (cos\theta_{i},sin\theta_{i})c & i=1,2,3,4\\ \sqrt{2}(cos\theta_{i},sin\theta_{i})c & i=5,6,7,8 \end{array}\right.$$

(2) $$\theta_{i}=\left\{\begin{array}{ll} \frac{\left(i-1\right)}{2}\pi & i=1,2,3,4\\ \frac{\left(i-5\right)}{2}\pi +\frac{\pi }{4} & i=5,6,7,8 \end{array}\right.$$

where *c* = Δ*x*/Δ*t*, Δ*x* is the lattice space and Δ*t* is the lattice time step size.

The mesoscopic momentum and heat distribution functions *f_i_*(***x***, *t*) and *g_i_*(***x***, *t*) can be obtained by solving LBEs:

(3) $$\left\{\begin{array}{l} f_{i}\left(x+c_{i}\Delta t,t+\Delta t\right)=f_{i}(x,t)\left(1-\omega_{f}\right)+\omega_{f}f_{i}^{eq}(x,t)+F\\ g_{i}\left(x+c_{i}\Delta t,t+\Delta t\right)=g_{i}(x,t)\left(1-\omega_{g}\right)+\omega_{g}g_{i}^{eq}(x,t) \end{array}\right.$$

where *ω*_f_ = Δ*t*/*τ*_f_ and *ω*_g_ = Δt/*τ*_g_ are the fluid relaxation factor and the thermal relaxation factor, *τ*_f_ and *τ*_g_ are the relaxation time of the velocity field and temperature field, and ***F*** is the external force of the particle, which is calculated by the Boussinesq approximation:

(4) F=ρgβΔT

where *ρ* is the density, g is the acceleration of gravity, *β* is the coefficient of thermal expansion, and Δ*T* is the difference between high and low temperature boundaries. The relaxation time for velocity and temperature fields is calculated as follows:

(5) $$\tau_{f}=2c_{s}^{2}\Delta t\left(2v+c_{s}^{2}\Delta t\right)=\frac{v}{c_{s}^{2}\Delta t}+\frac{1}{2}$$

(6) $$\tau_{g}=2c_{s}^{2}\Delta t\left(\frac{2\lambda }{\rho c_{p}}+c_{s}^{2}\Delta t\right)=\frac{\alpha }{c_{s}^{2}\Delta t}+\frac{1}{2}$$

where *v* is kinematic viscosity, *α* is the thermal diffusion rate, and *c*_s_ is the lattice sound velocity; for the D2Q9 model in this paper, it is $c_{s}^{2}$ = *c*^2^/3.

The equilibrium momentum and heat distribution functions are

(7) $$\left\{\begin{array}{l} f_{i}^{eq}=\omega_{i}\rho \left[1+\frac{3\left(c_{i}u\right)}{c^{2}}+\frac{9{\left(c_{i}u\right)}^{2}}{2c^{4}}-\frac{3u^{2}}{2c^{2}}\right]\\ g_{i}^{eq}=\omega_{i}T\left[1+\frac{3\left(c_{i}u\right)}{c^{2}}\right] \end{array}\right.$$

where ***u*** is the flow rate of the macroscopic insulating oil fluid.

The density, fluid flow velocity and temperature of the macroscopic insulating oil can be calculated by the sum of the momentum and heat balance functions along different lattice directions:

(8) $$\left\{\begin{array}{l} \rho =\sum_{i=0}^{8}f_{i}\\ u=\frac{1}{\rho }\sum_{i=0}^{8}c_{i}f_{i}\\ T=\sum_{i=0}^{8}g_{i} \end{array}\right.$$

### 3.2. Debris Motion Solution Based on DEM

When the DEM is used to calculate particle motion, it is necessary to analyze the force of different particle positions first. In the model constructed in this paper, the influence of pressure gradient force, Magnus force and Bassett force on particles are ignored, and only gravity ***F***_g_, buoyancy ***F***_b_, electric field force ***F***_e_ and oil flow drag force ***F***_d_ are considered. At this time, the motion equation of particles is as follows:

(9) $$m\frac{dv_{s}}{dt}=F_{g}+F_{b}+F_{e}+F_{d}$$

(10) $$\left\{\begin{array}{l} F_{g}=mg\\ F_{b}=\rho V_{p}g\\ F_{e}=0.83qE \end{array}\right.$$

(11) $$F_{d}=\frac{1}{8}\pi C_{D}\rho_{p}d_{p}^{2}\left|u-u_{p}\right|(u-u_{p})$$

where *m* is the particle mass, vs. is the particle velocity, *V*_p_ is the particle volume, *q* is the charge amount, ***E*** is the electric field strength, *C*_D_ is the fluid drag coefficient, *ρ*_p_ is the particle density, *d*_p_ is the particle diameter, and ***u***_p_ is the particle velocity.

In this study, the amorphous alloy core debris are flake-shaped, and their shape is quite different from that of spherical particles, so Formula (11) cannot be directly used to calculate the fluid drag force. The non-spherical particles were modified by introducing the equivalent spherical diameter *d*_p_ [23]:

(12) $$d_{p}={\left(\frac{6V_{p}}{\pi }\right)}^{\frac{1}{3}}$$

where *V*_p_ is the volume of the non-spherical particles.

Assuming that the cross-sectional diameter of the debris is *d* and the thickness is *h*, the diameter of the debris is calculated and substituted into Formula (12) to obtain the equivalent spherical diameter of the debris:

(13) $$d_{p}={\left(6dh\right)}^{\frac{1}{3}}$$

After the spherical correction of non-spherical debris, in the following calculations and descriptions, the debris are regarded as corrected spherical particles. In theory, the drag coefficient of fluid can be obtained from the Navier–Stokes equations of incompressible viscous fluid flowing around particles. However, due to the complexity of the floating surface layer on the particle surface, only a few special cases can be derived from the equations. Therefore, in this paper, the fluid drag coefficient is fitted with Reynolds number Re and sphericity ψ:

(14) $$Re=\frac{\rho d_{p}\left|u-u_{p}\right|}{\mu }$$

where *µ* is the dynamic viscosity of fluid.

The sphericity is defined as the ratio of the surface area of the sphere to the surface area Sp of the non-spherical particles, and the calculation formula is

(15) $$\psi =\frac{\pi d^{2}}{S_{p}}$$

Reference [24] analyzed the calculation results and experimental results of the empirical formula of the fluid drag coefficient proposed by different scholars and concluded that the empirical formula proposed by A. Haider and O. Levenspiel [25] had the smallest error:

(16) $$C_{D}=\frac{24}{Re}\left[1+ARe^{B}\right]+\frac{C\cdot Re}{D+Re}$$

(17) $$A=2.3288-6.4581\psi +2.4486\psi^{2}$$

(18) B=0.0964+0.55656ψ

(19) $$C=e^{4.905-13.8944\psi +18.4222\psi^{2}-10.2599\psi^{3}}$$

(20) $$D=e^{1.4681+12.2584\psi -20.7322\psi^{2}+15.8855\psi^{3}}$$

Because the motion of multiple particles may cause collisions between particles, each sub-step iteration needs to judge the collision of particles. In simulation, assuming that the centroid of particles is (*x_j_*, *y_j_*) and the number is *j*, the condition for collision between any two particles is

(21) $$\sqrt{{\left.\left(x_{j-1}-x_{j}\right.\right)}^{2}+{\left.\left(y_{j-1}-y_{j}\right.\right)}^{2}}\leq d_{p}\;j=2,3,4\text{...}$$

After particles collide, because the radius is the same, their charges will be transferred or neutralized, and finally the charges of the two are the same, and it is assumed that the collisions are completely elastic collisions; that is, the velocity remains unchanged, and the direction is opposite after the collision.

### 3.3. LBM–DEM Coupling

In order to consider the reaction force of particles on insulating oil during the solution process, it is necessary to couple its LBM and DEM, that is, to modify the LBM [26]. According to Newton’s third law, the force of particles on insulating oil is the reaction force of the drag force of oil flow, and an additional collision term can be introduced into the collision operator *Ω* of the LBM:

(22) $$\Omega =-\omega_{f}\left(1-B\right)\left[f_{i}(x,t)-f_{i}(x,t)\right]+B\Omega$$

(23) $$f_{i}(x,t+\Delta t)=f_{i}(x,t)-\omega_{f}\left(1-B\right)\cdot \left[f_{i}(x,t)-f_{i}^{eq}(x,t)\right]+B\Omega_{i}^{s}$$

(24) $$\Omega_{i}^{s}=\left[f_{-i}(x,t)-f_{-i}^{eq}(\rho ,u)\right]-\left[f_{i}(x,t)-f_{i}^{eq}(\rho ,u_{s})\right]$$

where −*i* and *i* have opposite directions, ***u*** and ***u***_s_ are the velocities of the fluid and solid at the node, respectively, and *B* is the weight of the additional collision term, calculated from the solid holdup *ε* at the node, as follows:

(25) $$B=\frac{\varepsilon \left(\omega_{f}-0.5\right)}{\left(1-\varepsilon \right)+\left(\omega_{f}-0.5\right)}$$

At this time, the oil drag force on the debris is related to the fluid velocity distribution, and the fluid velocity distribution is affected by the reaction force of the debris. Therefore, the LBM and DEM are coupled by the oil drag force.

### 3.4. Simulation Model and Physical Parameters

**Figure 5:**
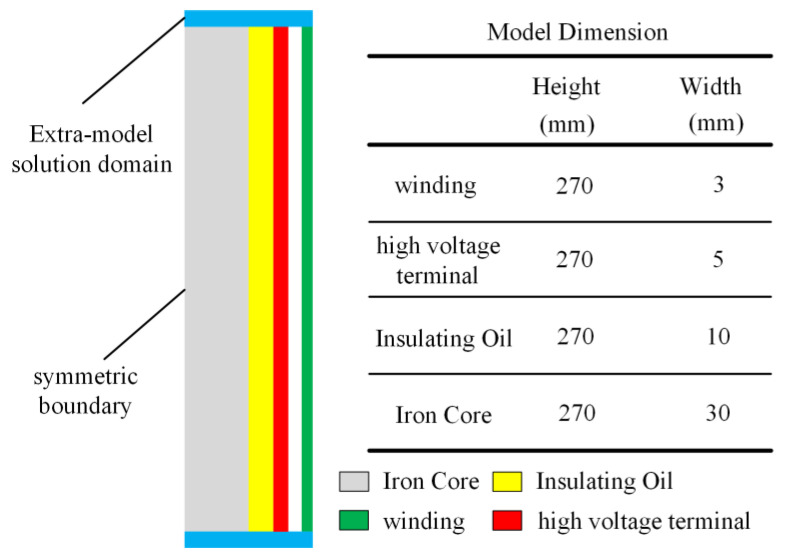
Simplified model of amorphous alloy transformer.

Most of the amorphous alloy core transformers adopt a three-phase four-frame five-column core structure. Each phase winding is sleeved on two adjacent frames with independent magnetic circuits and two adjacent frames in one winding. The third harmonic flux is numerically equal, and the phase is opposite. Therefore, the third harmonic flux component in each phase winding is zero, which reduces the eddy current loss caused by the third harmonic. Therefore, according to the symmetry of the three-phase winding, this paper selects the single-phase winding and its corresponding core column as the research object to select its XOY plane to establish a two-dimensional model. At the same time, the electromagnetic field inside each phase winding also has symmetry in the X direction. Therefore, one half of the XOY plane of the single-phase winding is selected to establish a two-dimensional plane calculation model, as shown in Figure 5.

In addition to the size of the above model, it is also necessary to determine the density *ρ*, thermal conductivity *k*, constant pressure specific heat *C*_p_ and kinematic viscosity *v* of the simulation model. The thermo-physical parameters of each part of the material are shown in Table 6.

## 4. Results and Discussion

### 4.1. Electric Field

**Figure 6:**
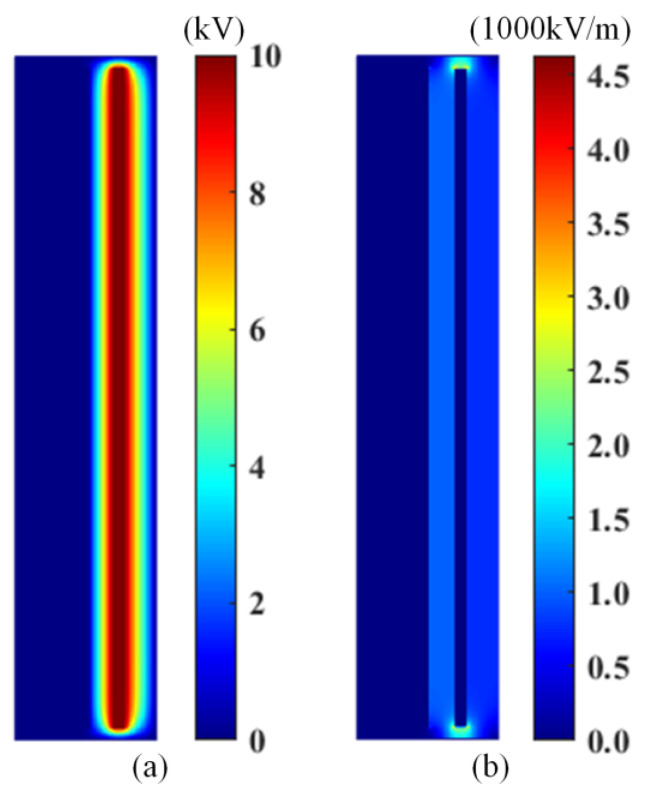
Potential distribution (**a**) and electric field distribution (**b**).

In the calculation of the electric field, the finite difference method is used to construct the same numerical model for calculation [27]. The grid side length is set to 0.5 mm. In order to obtain the electric field distribution at the upper and lower edges of the core and the electrode plate, the solution area is appropriately expanded at the upper and lower ends, and the grid size of the entire solution domain is set to 91 × 561, where the core is set to ground zero potential, and the high voltage electrode is set to 10 kV high potential. The distribution of potential and electric field intensity after solving is shown in Figure 6.

It can be seen from Figure 6 that the potential will be distorted at the boundary, but the total equipotential line is perpendicular to the horizontal line, so the electric field in the insulating oil gap can be approximately considered as a uniform electric field. The electric field intensity distribution in the figure also shows that the electric field intensity of the left insulating oil part is approximately 1000 kV/m, except that the electric field intensity near the upper and lower edges of the high voltage electrode is extremely large. After testing, we found that the electric field intensity shows a positive correlation with the change in voltage, so the different levels of alternating electric fields in this paper only need to consider the voltage frequency on the basis of the solved electric field to scale in equal proportion.

### 4.2. Thermal-Flow Field

**Figure 7:**
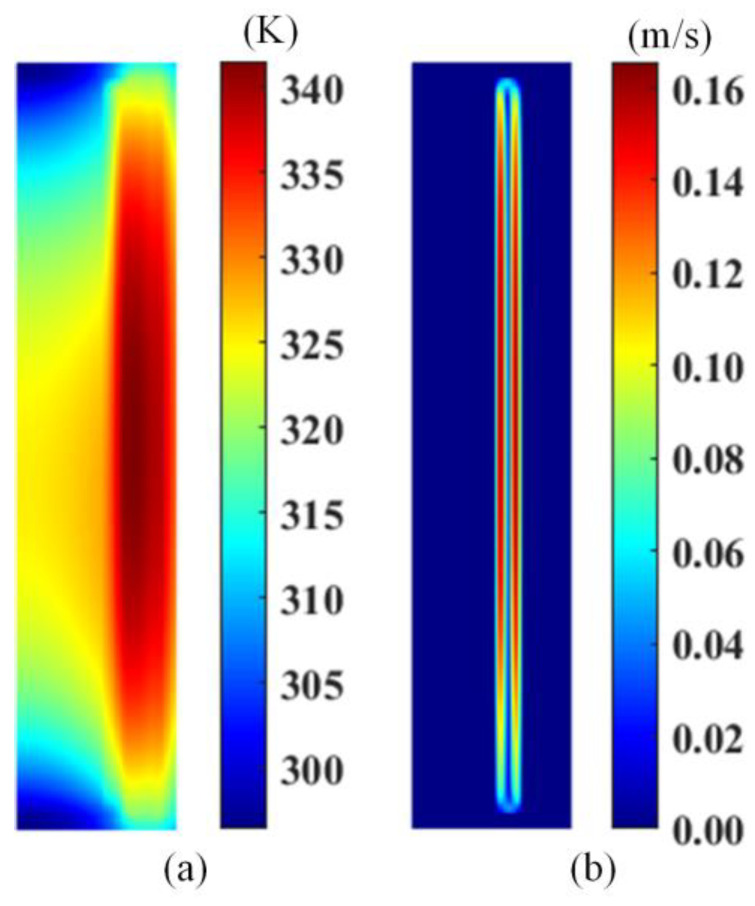
Temperature field (**a**) and velocity field (**b**).

According to the previous charging test, we set the initial conditions of the temperature field and the velocity field as the plate voltage of 6 kV. At this time, the steady-state thermal-flow field distribution obtained by the LBM–DEM simulation model is shown in Figure 7. At this time, the temperature field shows a decreasing trend from the middle high-voltage plate to both sides in the vertical direction. In the horizontal direction, the high-voltage electrode and winding are higher, and the temperature distribution of the core is the lowest. The temperature of all components shows a trend of high in the middle and low at both ends in the vertical direction. This is consistent with the law presented when the velocity field is stable, which is consistent with the natural convection phenomenon driven by non-isothermal oil flow. At this time, the maximum velocity of the velocity field is 0.16 m/s, and the oil flow direction is a counterclockwise natural convection trend.

### 4.3. Debris Migration Characteristics

**Figure 8:**
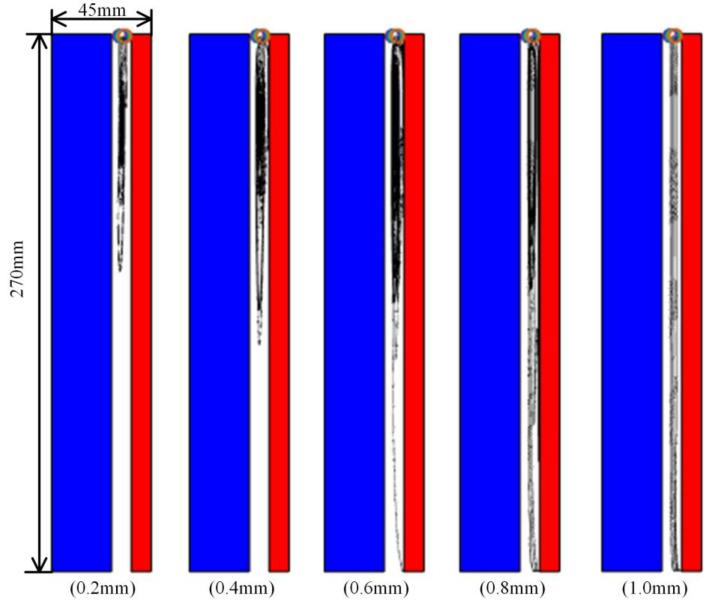
Motion trajectories of different debris sizes.

In the study of the migration characteristics of amorphous alloy core debris, the size of debris was set to 0.2 mm, 0.4 mm, 0.6 mm, 0.8 mm and 1.0 mm, respectively. The voltage was set to 6 kV, and the number of debris was four. The trajectory of debris after simulation is shown in Figure 8. The blue area in the figure is the core, the red area is the high-pressure electrode, the area between them is the insulating oil gap, the colored circle is the initial position of the particle, and the black curve is the trajectory of the particle.

It can be seen from the diagram that when the size of the amorphous alloy core debris is small, the trajectory of the debris is mostly concentrated on the upper end of the insulating oil gap of the amorphous alloy transformer and circulates with the natural convection direction of the insulating oil under the action of buoyancy and fluid drag force. When the radius of the debris increases, the effect of gravity begins to increase gradually. At this time, the trajectory of the debris will gradually move down. When the size exceeds 0.6 mm, there will be debris sinking to the end.

**Figure 9:**
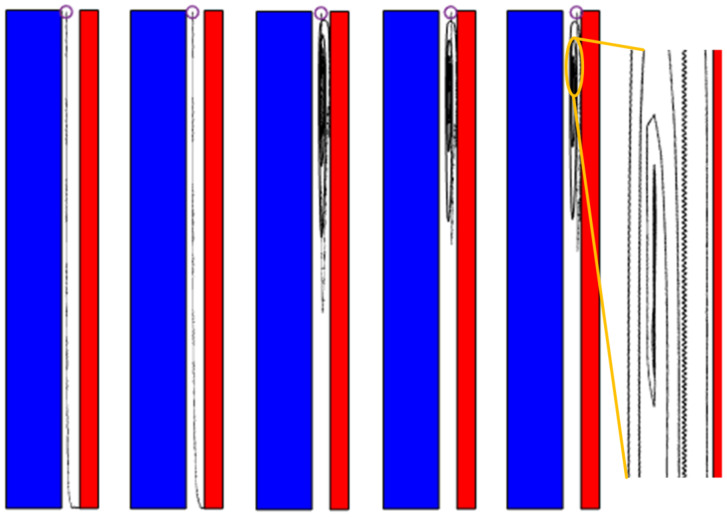
Motion trajectories of different initial positions.

In addition to the influence of the size of the debris on its trajectory, we note that the initial position of the debris will also affect its trajectory because this is related to the direction and size of the oil flow drag force that the debris is initially subjected to. Therefore, we carried out a study on the migration characteristics of debris at different initial positions of a single debris under the condition of voltage of 6 kV and debris size of 0.6 mm. The results are shown in Figure 9.

It can be seen from the diagram that when the initial debris position is on the left side, the natural convection direction of the insulating oil is counterclockwise. At this time, the gravity of the debris and the drag force of the oil flow are superimposed, and the debris accelerates to fall. When the debris reaches the bottom, the buoyancy and the oil flow drag force cannot change their velocity direction, which makes the debris sink to the bottom. When the initial position of the debris gradually moves to the right side, the oil flow drag force on the debris is upward, and the debris will move together with the natural convection direction of the insulating oil. This also explains that in the case of 0.6 mm in Figure 8, the particles on the left side sank to the bottom.

**Figure 10:**
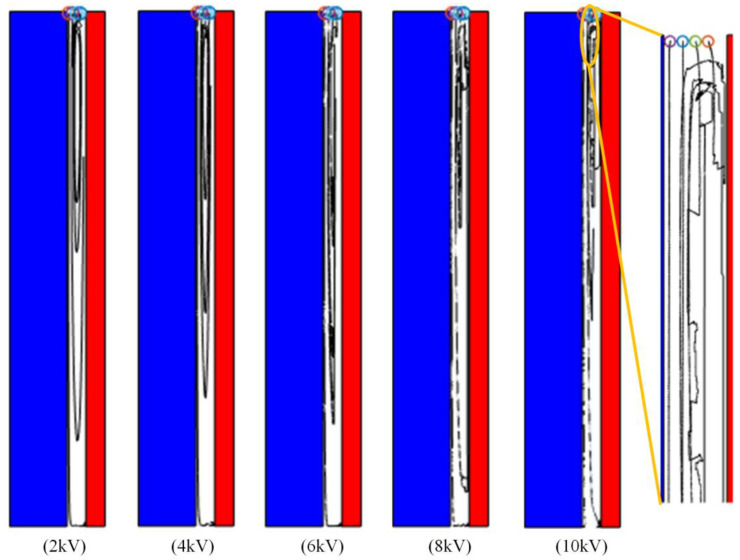
Motion trajectories of different voltage grades.

The size of the debris is kept at 0.6 mm, and the number is four. The electric field strength is changed to make the voltages 2 kV, 4 kV, 6 kV, 8 kV and 10 kV, respectively. At the same time, in order to reduce the impact of debris collision caused by the increase in voltage, the initial position is adjusted, and the initial spacing between debris is increased. The simulation results are shown in Figure 10. When the voltage is low, the debris circulates in the direction of the oil flow, and the trajectory is more regular, which is basically consistent with the direction of the insulating oil flow. When the voltage increases to 8 kV and 10 kV, the trajectory of the debris becomes irregular, and the lateral trajectory of the debris in the horizontal direction begins to increase significantly, and the probability of collision with the electrode also increases. The circulation of the debris with the flow of insulating oil is destroyed.

**Figure 11:**
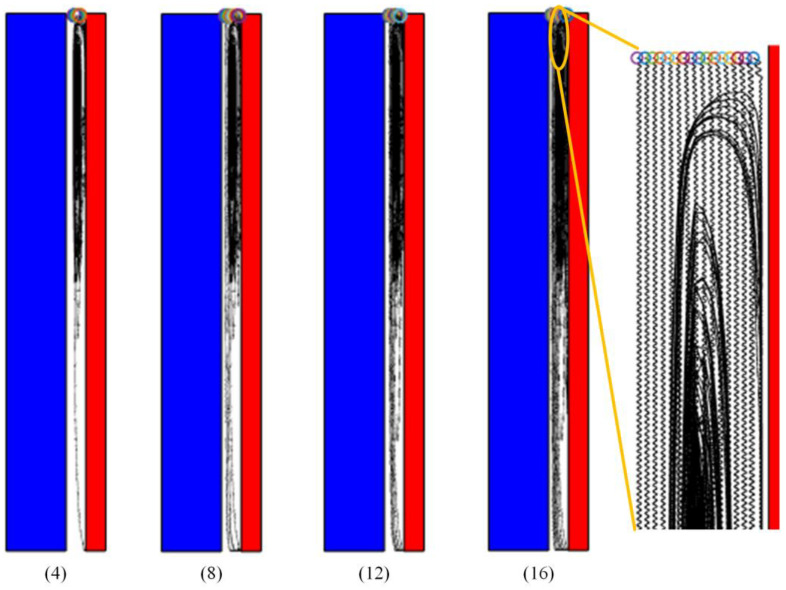
Motion trajectories of different debris numbers.

Finally, the probability of collision varies with the number of debris, and the transfer of charge and trajectory will also be affected. For this reason, we carried out simulation studies under the conditions of 6 kV voltage and 0.6 mm debris size, and the number of debris is 4, 8, 12 and 16, respectively. The results are shown in Figure 11. It can be seen that with an increase in the number of debris, some debris begin to move laterally or sink to the bottom, and the debris near the upper end of the high-pressure plate collide more.

It should be acknowledged that our two-dimensional model, while effective for investigating the motion characteristics of amorphous alloy core debris, possesses inherent limitations. Notably, it does not account for lateral debris movement or incorporate experimental validation through physical testing. In future studies, we intend to construct a physical test model of an amorphous alloy transformer and conduct systematic experiments based on the charging characteristics of the debris.

## 5. Conclusions

In this paper, a charging platform is set up, and a charging mechanism analysis of amorphous alloy core debris in insulating oil is realized. A two-dimensional amorphous alloy transformer calculation model based on the LBM–DEM is constructed, and the visualization of its internal thermal-flow field and the study of debris migration characteristics are realized. Therefore, it provides some reference for research on heat and mass transfer of amorphous alloy transformers.

(1) The physical test of charge characteristics of amorphous alloy core debris under an oil flow state is carried out. The test results show that with an increase in the insulating oil flow rate in the pipeline, the charge–mass ratio of debris will first increase and then decrease; with an increase in electric field strength, the charge-to-mass ratio of debris will gradually increase. As the number of debris increases, their charge-to-mass ratios will decrease; as the debris size increases, its charge-to-mass ratio will increase.
(2) The migration characteristics of amorphous alloy core debris under the thermal–fluidic–electrical coupling field of insulating oil were studied by the LBM–DEM model. The results show that when the size of the debris is small, the debris circulates with the natural convection direction of the insulating oil under the action of buoyancy and fluid drag. When the size of the debris increases, the effect of gravity is more obvious, and the debris will sink to the bottom. When there are fewer debris, it is not easy to collide with each other, which shows that they flow with the insulating oil. With an increase in the number of debris, the probability of collision between debris increases, the charge will be transferred, and some debris begin to move laterally or sink to the bottom. At the same time, the voltage intensity of the plate will also affect the movement of the debris. When the voltage is low, the debris is less affected by the electric field, and the trajectory is relatively regular, which is basically consistent with the flow direction of the insulating oil. As the electric field intensity in the oil gap increases, the trajectory of the debris becomes more and more irregular. At this time, the influence of the electric field force on the debris is increasing. Under the action of the AC electric field, the amplitude of the left and right back and forth oscillation is increasing, which interferes with the cyclic movement of the debris with the insulating oil.

## Figures and Tables

**Table 1:** Parameters of 1k101 Fe-based amorphous alloy strip.

Density/(kg/m^3^)	Saturation Magnetic Induction/T	Maximum Magnetic Permeability/(H/m)
7180	1.54	450,000

**Table 2:** Charge characteristics at different flow rates.

Flow Velocity/(m/s)	0.07	0.12	0.16	0.19	0.21
*q*/10^−10^ C	2.30	3.18	3.55	4.31	4.07
*Φ*/(10^−5^ C/kg)	3.42	4.73	5.28	6.41	6.05

**Table 3:** Charge characteristics under different voltages.

Voltage Level/kV	2	4	6	8	10
*q*/10^−10^ C	2.05	2.28	3.55	4.77	5.30
*Φ*/(10^−5^ C/kg)	3.05	3.39	5.28	7.09	7.88

**Table 4:** Charge characteristics under different numbers of particles.

Debris Number	4	8	12
*q*/10^−10^ C	3.55	6.41	8.68
*Φ*/(10^−5^ C/kg)	5.28	4.77	4.30

**Table 5:** Charge characteristics under different debris sizes.

Debris Size/mm	0.2	0.4	0.6	0.8	1.0
*q*/10^−10^ C	0.35	1.42	3.55	6.10	9.93
*Φ*/(10^−5^ C/kg)	4.69	4.75	5.28	5.10	5.32

**Table 6:** Thermo-physical properties of transformer components.

Item	Iron Core	Pole Plate/Winding	Insulating Oil [17]
*ρ*/kg·m^−3^	7 180	8 933	1098.72 − 0.712 *T*
*C_p_*/J·kg^−1^·K^−1^	540	381	807.163 + 3.58 *T*
*k*/W·m^−1^·K^−1^	10	387.6	0.159 − 7.101 × 10^−5^ *T*
*v*/kg·m^−1^·s^−1^	/	/	0.08467 − 0.000 4 *T* + 5 × 10^−7^ *T*^2^

## Data Availability

The original contributions presented in this study are included in the article. Further inquiries can be directed to the corresponding author.
